# Prevalence and Trends in Smoking Among Surgical Patients in Michigan, 2012-2019

**DOI:** 10.1001/jamanetworkopen.2021.0553

**Published:** 2021-03-03

**Authors:** Ryan Howard, Kushal Singh, Michael Englesbe

**Affiliations:** 1 Department of Surgery, University of Michigan, Ann Arbor; 2 Center for Healthcare Outcomes and Policy, University of Michigan, Ann Arbor; 3 Michigan Surgical Quality Collaborative, Ann Arbor

## Abstract

**Importance:**

Surgery is a teachable moment, and smoking cessation interventions that coincide with an episode of surgical care are especially effective. Implementing these interventions at a large scale requires understanding the prevalence and characteristics of smoking among surgical patients.

**Objectives:**

To describe the prevalence of smoking in a population of patients undergoing common surgical procedures and to identify any clinical or demographic characteristics associated with smoking.

**Design, Setting, and Participants:**

This cross-sectional study included all adult patients (aged ≥18 years) in a statewide registry who underwent general and vascular surgical procedures from 2012 to 2019 at 70 hospitals in Michigan. Data analysis was conducted from August to October 2020.

**Exposures:**

Undergoing a surgical procedure in any of the following categories: appendectomy, cholecystectomy, colon procedures, gastric or esophageal procedures, hepatopancreatobiliary procedures, hernia repair, small-bowel procedures, hysterectomy, vascular procedures, thyroidectomy, and other unspecific abdominal procedures.

**Main Outcomes and Measures:**

The prevalence of smoking prior to surgery, defined as cigarette use in the year prior to surgery, obtained from medical record review. Multivariable logistic regression was performed to analyze smoking prevalence based on insurance type and year of surgery while adjusting for demographic and clinical factors, including age, sex, race/ethnicity (determined from the medical record), insurance type, geographic region, comorbidities (ie, hypertension, diabetes, congestive heart failure, chronic obstructive pulmonary disease, chronic steroid use, and obstructive sleep apnea), American Society of Anesthesiologists classification, admission status, surgical priority, procedure type, and year of surgery.

**Results:**

From 2012 to 2019, 328 578 patients underwent surgery and were included in analysis. Mean (SD) age was 54.0 (17.0) years, and 197 501 patients (60.1%) were women. The overall prevalence of smoking was 24.1% (79 152 patients). Prevalence varied regionally from 21.5% (95% CI, 21.0%-21.9%; 6686 of 31 172 patients) in southeast Michigan to 28.0% (95% CI, 27.1%-28.9%; 2696 of 9614 patients) in northeast Michigan. When adjusting for clinical and demographic factors, there were greater odds of smoking among patients with Medicaid (odds ratio [OR], 2.75; 95% CI, 2.69-2.82) and patients without insurance (OR, 2.21; 95% CI, 2.10-2.33) compared with patients with private insurance. Among procedure categories, patients undergoing vascular surgery had greater odds of smoking (OR, 3.24; 95% CI, 3.11-3.38) than those undergoing cholecystectomy. Compared with 2012, the adjusted odds of smoking decreased significantly each year (eg, 2019: OR, 0.78; 95% CI, 0.74-0.81). In 2019, the adjusted prevalence of smoking was 22.3% (95% CI, 22.0%-22.7%) among all patients, 43.0% (95% CI, 42.4%-43.6%) among patients with Medicaid, and 36.3% (95% CI, 35.2%-37.4%) among patients without insurance.

**Conclusions and Relevance:**

In a statewide population of surgical patients, nearly one-quarter of patients smoked cigarettes, which is higher than the national average. The prevalence of smoking was especially high among patients without insurance and among those receiving Medicaid. Given the established association between undergoing a major surgical procedure and health behavior change, targeted smoking cessation interventions at the time of surgery may be an effective strategy to improve population health, especially among at-risk patient groups.

## Key Points

**Question:**

What is the prevalence of and what clinical and demographic characteristics are associated with smoking in a large population of patients undergoing surgery?

**Findings:**

In this cross-sectional study of 328 578 patients undergoing general and vascular surgical procedures from 2012 to 2019 in Michigan, nearly 1 in 4 patients smoked cigarettes at the time of surgery. Despite decreasing over the study period, in 2019 the adjusted prevalence of smoking was 22.3% among all patients, 43.0% among patients with Medicaid, and 36.3% among patients without insurance.

**Meaning:**

These findings indicate that smoking cessation interventions may be particularly important for patients undergoing surgery, especially for patients who lack health insurance or have Medicaid.

## Teaser

This cross-sectional study describes the prevalence of smoking in a population of patients undergoing common surgical procedures and identifies any clinical or demographic characteristics associated with smoking.

## Introduction

Despite significant progress in recent decades, smoking remains the leading preventable cause of death in the United States.^1^ The health care and societal costs associated with smoking exceed $320 billion annually.^2^ While smoking cessation programs are now widely available, more than 34 million adults still use tobacco, and less than 10% of individuals who smoke successfully quit each year.^3^ Effective mitigation of this public health crisis requires novel strategies. One such strategy is to leverage the unique nature of surgical care as an opportunity to achieve health behavior change.^4^ The notion of surgery as a transformative life event is intuitive, and the association between such an event and sustained behavior change is well-established.^5^ Patients undergoing surgery are especially receptive to behavior change.^6^ As such, surgery is a quintessential *teachable moment*, which is defined as an event that motivates individuals to spontaneously adopt risk-reducing health behaviors.^7,8^

While small-scale studies have demonstrated success in helping patients quit smoking around the time of surgery, the prevalence and characteristics of smoking across a large and diverse population of surgical patients is unknown.^9^ Surgical procedures and patient groups are often studied in isolation, and large-scale studies often do not include information about tobacco use at the time of surgery.^10,11,12^ For example, smoking prevalence can vary more than 2-fold depending on the population under analysis.^11,13^ In the general population, the prevalence of smoking also varies significantly based on insurance status, with individuals without insurance having a higher prevalence of smoking than those with private insurance.^14^ Identifying whether this trend translates to a surgical population is critical, given that a surgical episode may represent among the only interactions a patient without insurance has with the health care system and, therefore, an opportunity to improve health. Furthermore, although the prevalence of smoking has declined from 21% to 14% from 2009 to 2018, it is unknown whether this same trend has been observed in patients undergoing surgery.^1,3^ Given the effect of smoking on surgical outcomes as well as the unique role that surgical care may play in improving patients’ health behaviors, modern data are needed.

Therefore, in an effort to better understand opportunities to improve the long-term health behaviors of surgical patients, we investigated smoking prevalence and associated clinical and demographic characteristics in a large, regional population of patients undergoing surgery. We used data collected by the Michigan Surgical Quality Collaborative (MSQC) from 2012 to 2019. Our goal was to describe the prevalence of smoking in patients undergoing a variety of operations. Importantly, we sought to identify any significant trends among patient subgroups to better inform large-scale quality improvement efforts directed at achieving lasting smoking cessation after surgery.

## Methods

This cross-sectional study of deidentified secondary data was deemed exempt from review by the institutional review board of the University of Michigan. The requirement for informed consent was waived because of the lack of any identifying information. This study follows the Strengthening the Reporting of Observational Studies in Epidemiology (STROBE) reporting guideline.^15^

### Data Source and Patient Population

We conducted a retrospective analysis of data collected by the MSQC, a statewide collaborative quality improvement program that consists of 70 hospitals across the state of Michigan, representing all hospitals that perform major surgery.^16,17,18,19,20^ Of the 70 participating hospitals, 56 (80.0%) are located in metropolitan areas, 9 (12.9%) in micropolitan areas, and 5 (7.1%) in rural areas. Hospital sizes range from less than 300 beds in 42 hospitals (60.0%), 300 to 499 beds in 17 hospitals (24.3%), and 500 beds or more in 11 hospitals (15.7%). Eight hospitals (11.4%) are teaching-status hospitals. The MSQC uses a registry of prospectively collected data on patient demographic characteristics, perioperative processes, and 30-day outcomes for patients undergoing surgery.^21^ Participating hospitals receive funding from Blue Cross Blue Shield of Michigan to support trained Surgical Clinical Quality Reviewers (SCQR) who perform data abstraction. This abstraction of data relies on comprehensive review of a patient’s entire medical record by the SCQR, and interrater reliability assessments are regularly conducted to ensure validity and reliability of data. This process has advantages over using administrative claims in which miscoding or noncoding of diagnoses or procedures can compromise data validity.^22^ Cases are reviewed using a sampling algorithm designed to minimize selection bias, and data collection accuracy is audited annually.^23^

We included adult patients (aged ≥18 years) with complete registry data who underwent any surgery collected by the MSQC between January 1, 2012, and December 31, 2019. Patients were excluded if any explanatory variables were incomplete.

### Outcomes and Explanatory Variables

The primary outcome was the prevalence of smoking in the 12 months prior to surgery. This was ascertained from documentation in the medical record on review by the SCQR. This specifically referred to cigarette smoking and did not include electronic cigarettes (which contain no tobacco), marijuana, cigars, or chewing tobacco. The secondary outcomes were adjusted smoking prevalence by insurance type and by year of operation.

Demographic data included patient age, sex, race/ethnicity (determined by medical record review), insurance type, and geographic region. Insurance type was categorized into 5 primary groups, as follows: private, Medicare, Medicaid, uninsured, and other.^24^ Private insurance included any commercial health plan or a health maintenance organization (HMO) plan. Medicare included patients with Medicare, Medicare with a supplemental plan such as Medigap, or a Medicare Advantage plan. Medicaid included standard Medicaid or a Medicaid HMO plan. Uninsured patients had no active insurance at the time of surgery. The other category included non–Medicare or Medicaid government plans, such as Veterans Affairs or TriCare; self-pay with unspecified insurance; and other plans, such as worker’s compensation and automobile insurance.

To define and account for geographic variation, patients were grouped according to 10 prosperity regions, which each represent a unique and socioeconomically diverse local population.^25^ These 10 regions were the upper peninsula, northwest, northeast, west, east central, east, south central, southwest, southeast, and metropolitan Detroit. These regions have been used in previous work to examine variation in health behaviors and health care utilization in the state.^26^

Patient characteristics included diagnoses of hypertension, diabetes, congestive heart failure (CHF), chronic obstructive pulmonary disease (COPD), chronic steroid use, and obstructive sleep apnea (OSA). Clinical characteristics included American Society of Anesthesiologists (ASA) classification at the time of surgery, admission status (inpatient vs outpatient), surgical priority (elective vs urgent or emergent), year of surgery, and procedure category. Surgical procedures were grouped as follows: appendectomy, cholecystectomy, colon procedures, gastric or esophageal procedures, hepatopancreatobiliary procedures, hernia repair, small-bowel procedures, hysterectomy, vascular procedures, thyroidectomy, and other unspecified abdominal procedures.

### Statistical Analysis

Descriptive analysis was used to define the overall smoking prevalence among patients undergoing surgery as well as prevalence among subgroups based on explanatory variables. Univariate differences were calculated using the χ^2^ test. A multivariable logistic regression model was estimated to assess the prevalence of smoking while adjusting for relevant patient-level and clinical characteristics, including age, sex, race/ethnicity, insurance type, geographic region, hypertension, diabetes, CHF, COPD, chronic steroid use, OSA, ASA classification, admission status, surgical priority, procedure type, and year of surgery. Inclusion of age, sex, race/ethnicity, insurance type, and geographic region was based on the known association of each of these factors with the prevalence of smoking.^1,27,28,29^ Comorbidities and ASA classification were included based on the known association of tobacco use with various chronic illnesses, such as COPD, diabetes, and hypertension.^30,31^ Clinical factors, such as admission status, surgical priority, and procedure type, were included to adjust for differences in case mix between subgroups. Lastly, year of surgery was included as a covariate given that the overall prevalence of smoking declined during the study period.^1^ This model was used to calculate the smoking prevalence by insurance type from 2012 to 2019 while adjusting for these relevant demographic and clinical factors.

Statistical analyses were performed using Stata version 16.0 (StataCorp). *P* values were 2-tailed, and significance was set at *P* < .05. Multicollinearity was evaluated using variance inflation factors, and no significant multicollinearity was found for variables included in the model.

## Results

A total of 328 578 patients underwent surgery between 2012 and 2019 and were included in the MSQC registry. Mean (SD) age of the cohort was 54.0 (17.0) years, and 197 501 patients (60.1%) were women. Most patients were White individuals (271 675 [82.7%]) and lived in the metropolitan Detroit area at the time of their surgery (127 658 [38.9%]). Most patients had private insurance (161 529 [49.2%]), followed by Medicare (101 093 [30.8%]), Medicaid (50 507 [15.4%]), other insurance (7825 [2.4%]), and no insurance (7524 [2.3%]). The most common procedure was cholecystectomy (74 195 [22.6%]), followed by hernia repair (68 301 [20.8%]) and hysterectomy (54 925 [16.7%]). Complete descriptive statistics are displayed in Table 1.

**Table 1.** Baseline Demographic Characteristics and Univariate Statistics for Patients Undergoing Surgery Between 2012 and 2019

Characteristic	Overall, No. (% of column) (N = 328 578)	No. (% of row)		*P* value
		Smoked (n = 79 152)	Did not smoke (n = 249 426)	
Sex				
Male	131 077 (39.9)	33 714 (25.7)	97 363 (74.3)	<.001
Female	197 501 (60.1)	45 438 (23.0)	152 063 (77.0)	
Age, y				
<45	100 161 (30.5)	29 815 (29.8)	70 346 (70.2)	<.001
45-64	132 473 (40.3)	36 233 (27.4)	96 240 (72.7)	
>65	95 944 (29.2)	13 104 (13.7)	82 840 (86.3)	
Race/ethnicity				
White	271 675 (82.7)	64 268 (23.7)	207 407 (76.3)	<.001
Black	39 594 (12.1)	11 502 (29.1)	28 092 (70.9)	
American Indian or Alaskan Native	1200 (0.4)	420 (35.0)	780 (65.0)	
Native Hawaiian or Pacific Islander	223 (0.1)	36 (16.1)	187 (83.9)	
Asian	2570 (0.8)	233 (9.1)	2337 (90.9)	
Unknown	13 316 (4.1)	2693 (20.2)	10 623 (79.8)	
Region				
Upper peninsula	5646 (1.7)	1390 (24.6)	4256 (75.4)	<.001
Northwest	12 074 (3.7)	2861 (23.7)	9213 (76.3)	
Northeast	9614 (2.9)	2696 (28.0)	6918 (72.0)	
West	41 160 (12.5)	9252 (22.5)	31 908 (77.5)	
East central	26 629 (8.1)	6496 (24.4)	20 133 (75.6)	
East	38 919 (11.8)	10 529 (27.1)	28 390 (72.9)	
South central	11 359 (3.5)	2524 (22.2)	8835 (77.8)	
Southwest	24 347 (7.4)	6004 (24.7)	19 343 (75.4)	
Southeast	31 172 (9.5)	6686 (21.5)	24 486 (78.5)	
Metropolitan Detroit	127 658 (38.9)	30 714 (24.1)	96 944 (75.9)	
Insurance type				
Private	161 629 (49.2)	33 784 (20.9)	127 845 (79.1)	<.001
Medicaid	50 507 (15.4)	22 827 (45.2)	27 680 (54.8)	
Medicare	101 093 (30.8)	17 585 (17.4)	83 508 (82.6)	
Uninsured	7524 (2.3)	2955 (39.3)	4569 (60.7)	
Other	7825 (2.4)	2001 (25.6)	5824 (74.4)	
Comorbidities				
Hypertension	140 683 (42.8)	3022 (21.3)	110 661 (78.7)	<.001
Diabetes	46 201 (14.1)	9322 (20.2)	36 879 (79.8)	<.001
Congestive heart failure	2265 (0.7)	516 (22.8)	1749 (77.2)	.14
COPD	22 737 (6.9)	9418 (41.4)	13 319 (58.6)	<.001
Chronic steroid use	10 514 (3.2)	2179 (20.7)	8335 (79.3)	<.001
Obstructive sleep apnea	55 501 (16.9)	11 255 (20.3)	44 246 (79.7)	<.001
ASA classification, class				
1	24 935 (7.6)	2368 (9.5)	22 567 (90.5)	<.001
2	163 776 (49.8)	41 177 (25.1)	122 599 (74.9)	
3	121 224 (36.9)	30 681 (25.3)	90 543 (74.7)	
4	17 914 (5.5)	4721 (26.4)	13 193 (73.6)	
5	729 (0.2)	205 (28.1)	524 (71.9)	
Admission status				
Ambulatory	104 257 (31.7)	24 792 (23.8)	79 465 (76.2)	.005
Inpatient	224 321 (68.3)	54 360 (24.2)	169 961 (75.8)	
Surgical priority				
Elective	228 728 (69.6)	53 205 (23.3)	175 523 (76.7)	<.001
Urgent or emergent	99 850 (30.4)	25 947 (26.0)	73 903 (74.0)	
Procedure type				
Appendectomy	31 295 (9.5)	7973 (25.5)	23 322 (74.5)	<.001
Cholecystectomy	74 195 (22.6)	17 045 (23.0)	57 150 (77.0)	
Colon procedures	41 571 (12.7)	9567 (23.0)	32 004 (77.0)	
Gastric or esophageal procedures	11 584 (3.5)	2260 (19.5)	9324 (80.5)	
HPB procedures	3352 (1.0)	694 (20.7)	2658 (79.3)	
Hernia repair	68 301 (20.8)	16 083 (23.6)	52 218 (76.5)	
Small-bowel procedures	9526 (2.9)	2096 (22.0)	7430 (78.0)	
Hysterectomy	54 925 (16.7)	12 646 (23.0)	42 279 (77.0)	
Vascular procedures	22 108 (6.7)	8384 (37.9)	13 724 (62.1)	
Thyroidectomy	10 177 (3.1)	1974 (19.4)	8203 (80.6)	
Other abdominal procedures	1544 (0.5)	430 (27.9)	1114 (72.1)	
Year				
2012	16 123 (4.9)	4201 (26.1)	11 922 (73.9)	<.001
2013	37 389 (11.4)	9396 (25.1)	27 993 (74.9)	
2014	42 510 (12.9)	10 721 (25.2)	31 789 (74.8)	
2015	26 877 (8.2)	6884 (25.6)	19 993 (74.4)	
2016	48 084 (14.6)	11 980 (24.9)	36 104 (75.1)	
2017	54 040 (16.5)	12 933 (23.9)	41 107 (76.1)	
2018	43 900 (16.4)	12 254 (22.7)	41 646 (77.3)	
2019	49 655 (15.1)	10 783 (21.7)	38 872 (78.3)	

Abbreviations: ASA, American Society of Anesthesiologists; COPD, chronic obstructive pulmonary disease; HPB, hepatopancreatobiliary.

**Figure 1.**
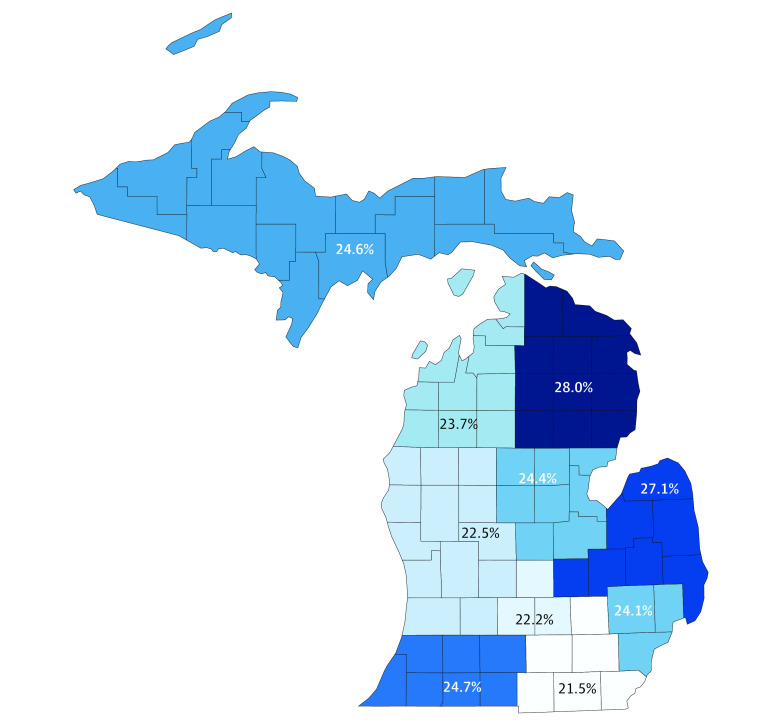
Geographic Variation in Unadjusted Smoking Prevalence Among Surgical Patients

The overall prevalence of smoking in this cohort was 24.1% (95% CI, 23.9%-24.2%; 79 152 patients). Univariate analysis revealed significant geographic variation in smoking prevalence, ranging from 21.5% (95% CI, 21.0%-21.9%; 6686 of 31 172 patients) in southeast Michigan to 28.0% (95% CI, 27.1%-28.9%; 2696 of 9614 patients) in northeast Michigan (Figure 1). The prevalence of smoking was significantly higher among patients with Medicaid (22 827 [45.2%; 95% CI, 44.8%-45.6%]) and patients without insurance (2955 [39.3%; 95% CI, 38.2%-40.4%]) compared with patients with private insurance (33 784 [20.9%; 95% CI, 20.7%-21.1%]) or patients with Medicare (17 585 [17.4%; 95% CI, 17.2%-17.6%]) (*P* < .001). Among the included comorbidities, the prevalence of smoking was highest among patients with COPD (9418 of 22 737 [41.4%]; *P* < .001). The prevalence of smoking was also significantly higher among patients undergoing vascular surgery (8384 of 22 108 [37.9%; *P* < .001) compared with other surgical procedures. The unadjusted prevalence of smoking declined significantly from 26.1% (4201 of 16 123) in 2012 to 21.7% (10 783 of 49 655) in 2019 (*P* < .001).

**Figure 2.**
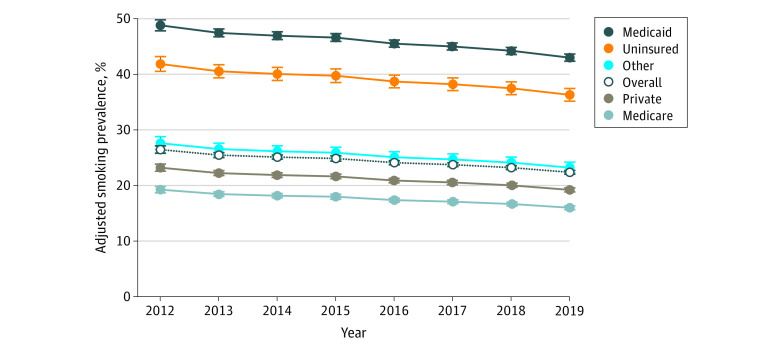
Risk-Adjusted Smoking Prevalence by Insurance Type, 2012-2019 Error bars indicate 95% CIs.

Results of the multivariable logistic regression are displayed in Table 2. After controlling for important demographic and clinical factors, patients with Medicaid had higher odds of smoking (odds ratio [OR], 2.75; 95% CI, 2.69-2.82), as did patients without insurance (OR, 2.21; 95% CI, 2.10-2.33), patients with Medicare (OR, 1.48; 95% CI, 1.43-1.53), and patients with other insurance (OR, 1.32; 95% CI, 1.25-1.39). There were also higher odds of smoking among patients with more serious ASA classifications, with COPD, undergoing ambulatory procedures, and undergoing urgent or emergent surgery. Female sex was associated with lower odds of smoking (OR, 0.79; 95% CI, 0.77-0.80). Compared with patients younger than 45 years, patients aged 45 to 64 years had lower odds of smoking (OR, 0.79; 95% CI, 0.77-0.81), as did patients aged 65 years and older (OR, 0.22; 95% CI, 0.21-0.23). Among procedure categories, patients undergoing vascular surgery had the highest odds of smoking (OR, 3.26; 95% CI, 3.13-3.39). Compared with 2012, the adjusted odds of smoking in this cohort decreased significantly each year but varied by insurance type (eg, 2019: OR, 0.78; 95% CI, 0.74-0.81). In 2019, the adjusted prevalence of smoking was 22.3% (95% CI, 22.0-22.7%) among all patients, 43.0% (95% CI, 42.4%-43.6%) among patients with Medicaid, and 36.3% (95% CI, 35.2%-37.4%) among patients without insurance (Figure 2).

**Table 2.** Multivariable Logistic Regression for Clinical and Demographic Characteristics Associated With Smoking at the Time of Surgery

Characteristic	OR (95% CI)	*P* value
Sex		
Male	1 [Reference]	NA
Female	0.79 (0.77-0.80)	<.001
Age, y		
<45	1 [Reference]	NA
45-64	0.79 (0.77-0.81)	<.001
≥65	0.22 (0.21-0.23)	<.001
Race/ethnicity		
White	1 [Reference]	NA
Black	1.00 (0.97-1.03)	.90
American Indian or Alaskan Native	1.44 (1.26-1.64)	<.001
Native Hawaiian or Pacific Islander	0.52 (0.35-0.77)	.001
Asian	0.35 (0.31-0.41)	<.001
Unknown	0.72 (0.69-0.76)	<.001
Insurance type		
Private	1 [Reference]	NA
Medicaid	2.75 (2.69-2.82)	<.001
Medicare	1.48 (1.43-1.53)	<.001
Uninsured	2.21 (2.10-2.33)	<.001
Other	1.32 (1.25-1.39)	<.001
Region		
Metropolitan Detroit	1 [Reference]	NA
Upper peninsula	1.06 (0.99-1.13)	.09
Northwest	1.05 (1.00-1.10)	.03
Northeast	1.20 (1.15-1.27)	<.001
West	0.91 (0.88-0.93)	<.001
East central	1.00 (0.96-1.03)	.87
East	1.13 (1.10-1.16)	<.001
South central	0.91 (0.86-0.95)	<.001
Southwest	0.98 (0.95-1.01)	.24
Southeast	0.90 (0.87-0.93)	<.001
Comorbidities		
Hypertension	0.81 (0.79-0.83)	<.001
Diabetes	0.71 (0.69-0.73)	<.001
Congestive heart failure	0.82 (0.74-0.92)	<.001
COPD	2.83 (2.73-2.92)	<.001
Chronic steroid use	0.69 (0.66-0.73)	<.001
Obstructive sleep apnea	0.77 (0.75-0.79)	<.001
ASA classification, class		
1	1 [Reference]	NA
2	4.07 (3.89-4.26)	<.001
3	5.18 (4.93-5.44)	<.001
4	4.84 (4.54-5.16)	<.001
5	4.90 (4.04-5.93)	<.001
Admission status		
Inpatient	1 [Reference]	NA
Ambulatory	1.15 (1.13-1.18)	<.001
Surgical priority		
Elective	1 [Reference]	NA
Urgent or emergent	1.16 (1.13-1.20)	<.001
Procedure type		
Cholecystectomy	1 [Reference]	NA
Appendectomy	1.11 (1.07-1.15)	<.001
Colon procedures	1.35 (1.30-1.40)	<.001
Gastric or esophageal procedures	0.97 (0.92-1.02)	.28
HPB procedures	1.26 (1.15-1.38)	<.001
Hernia repair	1.20 (1.16-1.23)	<.001
Small-bowel procedures	1.17 (1.11-1.24)	<.001
Hysterectomy	1.16 (1.12-1.20)	<.001
Vascular procedures	3.24 (3.11-3.38)	<.001
Thyroidectomy	0.97 (0.92-1.03)	.31
Other abdominal procedures	1.43 (1.27-1.63)	<.001
Year		
2012	1 [Reference]	NA
2013	0.94 (0.90-0.99)	.01
2014	0.92 (0.88-0.96)	<.001
2015	0.91 (0.87-0.95)	<.001
2016	0.87 (0.83-0.91)	<.001
2017	0.85 (0.81-0.89)	<.001
2018	0.82 (0.79-0.86)	<.001
2019	0.78 (0.74-0.81)	<.001

Abbreviations: ASA, American Society of Anesthesiologists; COPD, chronic obstructive pulmonary disease; HPB, hepatopancreatobiliary; OR, odds ratio; NA, not applicable.

## Discussion

In this population-based study of patients undergoing surgery in Michigan, nearly 1 in every 4 patients smoked cigarettes at the time of surgery. There was significant variation in the prevalence of smoking by patient characteristics, geographic region, insurance status, and procedure type. Despite a gradual decline in smoking prevalence during the study period, patients with Medicaid and those without insurance continued to have nearly twice the prevalence of smoking as recently as 2019 compared with patients with private insurance or Medicare. Recognizing these trends in smoking prevalence among surgical patients is the first step to designing interventions to achieve sustained smoking cessation after surgery.

An important finding of this study is that the prevalence of smoking among surgical patients is higher than that of the general population. In 2018, 18.9% of adults in Michigan smoked cigarettes.^32^ However, in this cohort of patients undergoing surgery, risk-adjusted smoking prevalence that same year was 22.8%. This is also considerably higher than that of the general population of the United States, where smoking prevalence was 13.8% in 2018.^3^ Similarly, the most recent Behavioral Risk Factor Survey found that the prevalence of smoking among individuals without insurance in Michigan was 34.2% in 2017 compared with a prevalence of 38.0% in our cohort of surgical patients in the same year.^33^ Although smoking prevalence among patients with Medicaid in Michigan has not been described, nationally it was 23.9% in 2018 compared with 43.8% in our cohort in the same year.^3^

The finding that smoking prevalence is higher among surgical patients, especially among patients without insurance and those receiving Medicaid, represents an important opportunity to intervene on behavioral risk around the time of surgery. For a patient without insurance, an unplanned surgical episode may be among the only interactions they have with the health care system. Therefore, policies or interventions that enable sustained behavior change may be particularly important to this group, who lack access to the resources commonly required to quit smoking and are therefore less likely to quit than patients with health insurance.^34^ Others have similarly suggested leveraging a trauma episode as a potential entry point to receiving comprehensive health care for populations with higher risk.^35^ Patients with Medicaid also face significant barriers to accessing smoking cessation resources. Although state Medicaid coverage for smoking cessation treatments has become more expansive since the passage of the Patient Protection and Affordable Care Act, as of 2019 only 13 states had comprehensive coverage for smoking cessation treatments.^36^ Medicaid enrollees are less likely to successfully navigate the health care system to obtain smoking cessation assistance, and only 10% of enrollees who smoked in 2013 received a prescription for a tobacco cessation medication.^28,37^ As a result, the prevalence of smoking among Medicaid patients has declined at a slower rate than among the general population during the last decade.^38^ Similarly among age groups, our study found that the prevalence of smoking was highest among younger patients, for whom smoking cessation may have even greater long-term health benefits than among older adults.^39^

A surgical episode may be a particularly effective time to engage patients in health behavior change if traditional methods have failed. Antitobacco public health campaigns, smoking cessation resources, and regulations to prevent passive smoke exposure are now widely prevalent throughout the United States, yet the annual quit rate among smokers is only 7.5%.^3^ Moreover, these efforts compete with a tobacco industry that spends $25 million each day to promote cigarettes and smokeless tobacco products.^40^ Surgery may represent a unique opportunity to augment these efforts. US residents undergo an average of 9 surgical procedures in their lifetime.^41^ Major medical events or new diagnoses greatly increase the likelihood that a patient will adopt lasting healthy behaviors.^4^ For example, while less than 10% of individuals who smoke spontaneously quit smoking each year, more than 50% of patients undergoing surgery for smoking-related diseases successfully quit after surgery.^9,42^ Even patients undergoing surgery for non–smoking-related diseases, such as orthopedic surgery, are more likely to quit smoking.^43^ While surgeons recognize the importance of health behaviors, such as smoking, most do not engage their patients in these domains, citing time constraints, resource limitations, or a belief that such efforts would simply be futile.^44,45^ When smoking is addressed, it is typically in the setting of prehabilitation, with the goal of cessation prior to surgery rather than after surgery.^46^

Taking advantage of surgery as a teachable moment to achieve health behavior change would increase the value of surgical care to society.^47^ Despite significant improvement in surgical outcomes, such as mortality and complication rates, surgery has only a modest impact on the overall health of society, which is predominantly driven by health behaviors.^47,48^ Behavioral factors, such as smoking, physical activity, and diet, make up 9 of the top 10 risk factors for death and disability and account for nearly half of all premature deaths in the United States.^49,50,51^ Surgical care, on the other hand, prevents less than 10% of premature mortality in this country.^48^ As an illustration, a patient who undergoes a laparoscopic cholecystectomy for acute cholecystitis costs the health care system $6000.^52,53^ However, if that same patient smokes, even with a perfect surgical outcome, they will go on to cost the health care system $32 000 in the following year and $1.6 million in their lifetime.^54^ Currently, no metrics exist to quantify the success or failure of affecting health behaviors as part of surgical care.

If high-value surgical care is to include smoking cessation, this paradigm change must begin with the surgeon. Raising surgeon awareness and changing practice by engaging patients in health behavior change is a necessary centerpiece to this work. To that end, surgeons should make an active effort to counsel patients about smoking cessation and connect them with the best available resources. It has been shown that surgeons generally underestimate the information that patients are receptive to during a preoperative consultation.^55^ Therefore, expanding counseling to include guidance on improving health behaviors and quitting smoking may be especially impactful. Capitalizing on behavior change around the time of surgery requires a multifaceted strategy that engages many stakeholders. Professional organizations can play a central role in these efforts by including perioperative smoking cessation as part of their agenda. For example, the American College of Surgeons offers a Quit Smoking Before Surgery program to help surgeons implement smoking cessation services into their clinical workflow.^56^ Similarly, the MSQC itself has a track record of guiding statewide quality improvement efforts in Michigan and is currently in the process of leveraging its clinical registry to help patients improve health behaviors around the time of surgery. In this case, intervention may be as pragmatic as connecting every surgical patient who smokes with tobacco cessation services, which are widely available at all health systems but significantly underutilized at the time of surgery.^57^ Alternatively, in the United Kingdom, the Make Every Contact Count program leverages every interaction a patient has with the health care system as an opportunity to positively change health behaviors.^58,59^ Some have even suggested redesigning the surgical pathway to integrate health behavior screening and engagement as a standard practice at multiple points.^60^ Currently in Michigan, perioperative health behavior screenings and interventions are being piloted within the MSQC.

### Limitations

This study has limitations. First, the intent of this study was to describe the prevalence of and trends in tobacco use at the time of surgery, and therefore, no information was collected regarding smoking cessation interventions or quit rates after surgery. The purpose of this study was to assess the current state of tobacco use at the time of surgery, and future work is critically needed to assess the actual effect of smoking cessation interventions among surgical patients. Current efforts are under way in Michigan to collect long-term smoking outcomes after surgery. Although the study sample drew from a diverse population of surgical patients, selection bias exists given the retrospective nature of this study. Moreover, smoking prevalence varies significantly between states, and the results demonstrated in the population of our state may not be generalizable to other states where there are well-documented differences in the overall prevalence of smoking. Nevertheless, in comparing smoking prevalence to the general population, we used statistics specific to the state of Michigan to ensure a meaningful comparison. Another limitation of this study is that although employment status, occupation, education, and household income have been shown to be highly associated with smoking prevalence, the MSQC database does not capture this information, and therefore, we were unable to include it in analysis.^61^ Efforts to link these critical demographic characteristics to smoking prevalence and outcomes are currently under way in Michigan as the MSQC increases its focus on addressing this public health problem in patients undergoing surgery. The MSQC also does not capture data regarding passive smoking exposure, which is another major driver of premature morbidity and mortality.^62^ This cohort was limited to patients undergoing a variety of general and vascular surgical procedures and did not include patients undergoing specialized procedures in subspecialties such as orthopedics, cardiac surgery, or otolaryngology, where smoking prevalence likely differs as well. However, the procedures included in this analysis are some of the most common performed in the United States. Furthermore, although our study describes the prevalence of and factors associated with smoking among patients undergoing surgery, it does not offer any information about how best to mitigate smoking in this population. Future work is needed to investigate the efficacy of targeted interventions now that the characteristics of smoking in this population have been described.

## Conclusions

In a statewide population of surgical patients, nearly one-quarter of patients smoked cigarettes, which is higher than the national average. The prevalence of smoking was especially high among patients without insurance and those receiving Medicaid even as recently as 2019. Given the established association between undergoing a major surgical procedure and health behavior change, targeted smoking cessation interventions at the time of surgery may be an effective strategy to improve population health, especially among patient groups with high risk.
